# Transcriptomic Analysis of Circulating Leukocytes Obtained during the Recovery from Clinical Mastitis Caused by *Escherichia coli* in Holstein Dairy Cows

**DOI:** 10.3390/ani12162146

**Published:** 2022-08-21

**Authors:** Zhangrui Cheng, Sergio Palma-Vera, Laura Buggiotti, Mazdak Salavati, Frank Becker, Dirk Werling, D. Claire Wathes, GplusE Consortium

**Affiliations:** 1 Department for Pathobiology and Population Sciences, Royal Veterinary College, Hatfield AL9 7TA, UK; 2 Research Institute for Farm Animal Biology, Wilhelm-Stahl-Allee 2, 18196 Dummerstorf, Germany; 3 Centre for Vaccinology and Regenerative Medicine, Royal Veterinary College, Hatfield AL9 7TA, UK

**Keywords:** *E. coli* mastitis, cow, mammary gland, antimicrobial peptides, ABC transporters, MHC system

## Abstract

**Simple Summary:**

*Escherichia coli* is a bacterium which infects cow udders causing clinical mastitis, a potentially severe disease with welfare and economic consequences. During an infection, white blood cells (leukocytes) enter the udder to provide immune defence and assist tissue repair. We sequenced RNA derived from circulating leukocytes to investigate which genes are up- or down-regulated in dairy cows with naturally occurring cases of clinical mastitis in comparison with healthy control cows from the same farm. We also looked for genetic variations between infected and healthy cows. Blood samples were taken either EARLY (around 10 days) or LATE (after 4 weeks) during the recovery phase after diagnosis. Many genes (1090) with immune and inflammatory functions were up-regulated during the EARLY phase. By the LATE phase only 29 genes were up-regulated including six haemoglobin subunits, possibly important for the production of new red blood corpuscles. Twelve genetic variations which were associated with an increased or decreased expression of some important immune genes were identified between the infected and control cows. These results show that the initial inflammatory response to *E. coli* continued for at least 10 days despite the cows having received prompt veterinary treatment, but they had largely recovered within 4 weeks. Genetic differences between cows may predispose some animals to infection.

**Abstract:**

The risk and severity of clinical infection with *Escherichia coli* as a causative pathogen for bovine mastitis is influenced by the hosts’ phenotypic and genotypic variables. We used RNA-Seq analysis of circulating leukocytes to investigate global transcriptomic profiles and genetic variants from Holstein cows with naturally occurring cases of clinical mastitis, diagnosed using clinical symptoms and milk microbiology. Healthy lactation-matched cows served as controls (CONT, *n* = 6). Blood samples were collected at two time periods during the recovery phase post diagnosis: EARLY (10.3 ± 1.8 days, *n* = 6) and LATE (46.7 ± 11 days, *n* = 3). Differentially expressed genes (DEGs) between the groups were identified using CLC Genomics Workbench V21 and subjected to enrichment analysis. Variant calling was performed following GATKv3.8 best practice. The comparison of *E. coli*(+) EARLY and CONT cows found the up-regulation of 1090 DEGs, mainly with immune and inflammatory functions. The key signalling pathways involved NOD-like and interleukin-1 receptors and chemokines. Many up-regulated DEGs encoded antimicrobial peptides including cathelicidins, beta-defensins, S100 calcium binding proteins, haptoglobin and lactoferrin. Inflammation had largely resolved in the *E. coli*(+) LATE group, with only 29 up-regulated DEGs. Both EARLY and LATE cows had up-regulated DEGs encoding ATP binding cassette (ABC) transporters and haemoglobin subunits were also up-regulated in LATE cows. Twelve candidate genetic variants were identified in DEGs between the infected and CONT cows. Three were in contiguous genes *WIPI1, ARSG* and *SLC16A6* on BTA19. Two others (*RAC2* and *ARHGAP26)* encode a Rho-family GTPase and Rho GTPase-activating protein 26. These results show that the initial inflammatory response to *E. coli* continued for at least 10 days despite prompt treatment and provide preliminary evidence for genetic differences between cows that may predispose them to infection.

## 1. Introduction

Mastitis is an inflammatory condition of the mammary gland which causes significant economic losses due to the cost of treatment, reduced milk production, discarding milk and the death or culling of infected cows [1]. *Escherichia coli* (*E. coli*) infection is generally associated with the rapid onset of acute mastitis, sometimes with severe systemic clinical symptoms including pyrexia, diarrhoea and dehydration, which have an adverse effect on animal welfare and may cause mortality [2]. The bacterial strain, cow genotype and physiological state and the farm environment can all interdependently affect mastitis susceptibility [3,4]. Studies have shown that the severity of *E. coli* mastitis is predominantly determined by cow factors rather than by *E. coli* pathogenicity [5,6], although some *E. coli* strains are better able to invade and replicate within mammary epithelial cells and can therefore cause a persistent intramammary infection [7].

The cell wall component lipopolysaccharide (LPS) is the main pathogen-associated molecular pattern (PAMP) for *E. coli* [6]. The LPS released within the mammary gland is recognised by the LPS receptor complex composed of the LPS-binding protein, Toll-like receptor (TLR) 4, myeloid differentiation protein 2 (MD-2) and cluster of differentiation (CD) 14. This recognition leads to the cascade activation of NFKB and other transcription factor pathways which induce a rapid and strong rise in the expression of various pro-inflammatory genes encoding cytokines, chemokines, prostaglandins and adhesion molecules which activate the cells of the innate and adaptive immune systems [8,9,10,11]. These signalling pathways also promote the production of oxygen and nitric oxide radicals and anti-inflammatory cytokines such as IL-10 and TGFß [9]. The initial response to LPS leads to the recruitment of circulating leukocytes, especially neutrophils, to the inflamed mammary gland where they play crucial roles in the initiation, development and resolution of mastitis [12]. In order for the individual cow to effectively deal with *E. coli* mastitis, the movement of leukocytes into the mammary gland must occur in a timely fashion and be properly controlled [13]. A mild response may fail to achieve pathogen elimination whereas an excessive and prolonged response is more likely to cause additional immune-response-induced tissue damage [14].

The majority of previous studies using transcriptomic profiles to investigate the response to mastitis-causing infections have analysed mammary gland biopsies or leukocytes collected from cows in which the infection was experimentally induced by intra-mammary inoculation with *E. coli* [6,15,16,17] or LPS [18]. Much less information is available concerning naturally occurring clinical cases. In the present study, we investigated changes in global transcriptomic profiles of circulating leukocyte in mid-lactation cows on a single farm experiencing clinical mastitis caused by *E. coli* using next-generation RNA sequencing and bioinformatics approaches. RNA-Seq-derived variants were also compared between the infected individuals and healthy control cows. The aims were firstly to understand more about how naturally infected cows continue to respond to the infection during the resolution phase and secondly to investigate whether there were any genetic differences between animals which are more or less susceptible to infection.

## 2. Materials and Methods

### 2.1. Animals

Suitable Holstein cows were recruited from a single 800 cow commercial dairy farm located in Mecklenburg-Pomerania, Germany, with an average 305 d milk yield exceeding 10,000 kg. All recruited cows were sampled in a single three-month summer period from May to July. All procedures were approved by the Animal Welfare Committee of Mecklenburg-Pomerania/Germany (LALLF 7221.3-18196-22-03). Ten cows with suspected cases of *E. coli* mastitis were examined by the attending veterinarian. One cow had no clinical symptoms and so was excluded from the study. Milk samples were taken before medical treatment and quick on-farm microbiological tests were performed. Additional milk samples were taken for submission to an approved Central Laboratory for microbiological testing (see below). All cows with severe symptoms (*n* = 8) were locally and systemically treated immediately with an aminoglykoside antibiotic and Metacam^®^, a nonsteroidal anti-inflammatory drug (NSAID) (Boehringer Ingelheim, Ingelheim am Rhein, Germany). In a few cases *(n* = 3), water drenching and stimulation of the rumen were also essential. Refer to Supplementary Table S1 for further cow details. Nine control *E. Coli*(−) cows (CONT) were recruited from the same farm and were matched as closely as possible with respect to lactation number, date and days in milk. The controls were therefore exposed to the same environmental conditions as the *E. col*i(+) cows. Milk samples were also taken from the control cows to exclude sub-clinical mastitis. The microbiological tests showed that three of them were infected with *Streptococci* or other bacteria. These cows were therefore excluded from analysis of the differential gene expression and the *in vitro* tests.

Blood samples were taken during a routine veterinary examination performed at a median time of 15 days (range 6–65 days) after the initial diagnosis. Heparinised fresh blood (15 mL) from each cow was collected by jugular venepuncture into a Falcon tube for in vitro testing and a second blood sample was taken into a Tempus blood collection tube (Thermo Fischer Scientific, Loughborough, UK), which was shaken vigorously for 15–20 s immediately upon collection, then frozen and stored at −80 °C for subsequent RNA extraction.

### 2.2. Microbiological Analysis

All milk samples were sent immediately to an approved Central Diagnostic Laboratory in Mecklenburg–Pomerania, Germany, for analysis by matrix-assisted laser desorption/ionisation time-of-flight mass spectrometry (MS), an approved routine method to identify specific microorganisms. Firstly, samples were inoculated in blood agar (BA; Caseinpepton-soya flour pepton-agar enriched with 5% blood from sheep), using a sterile swab. After inoculation, the BA plates were incubated at 37 °C for 48 h. Afterwards, colonies of the samples were selected for microbiological identification by MS. For protein extraction, a colony was selected from each isolate and applied to a steel plate containing 96 wells, prepared for identification by the MALDITOF Biotyper (MSP 96 Target polished steel, Bruker Daltonik GmbH, Bremen, Germany).

### 2.3. In Vitro Blood Tests

The fresh blood samples were taken to the laboratory at the Research Institute for Farm Animal Biology, layered onto Histopaque (Sigma-Aldrich Chemie GmbH, Taufkirchen, Germany) and centrifuged at 1200× *g* for 30 min after which the opaque layer of peripheral blood mononuclear cells (PBMCs) was aspirated from the plasma/histopaque interface. These were washed ×3 in phosphate-buffered saline, counted and re-suspended in medium (RPMI 1640 with 10% FBS, 1% Glutamax, 1% pen/strep (10,000 U/mL penicillin, 10,000 µg/mL streptomycin)) to a concentration of 1 × 10^6^ cells/mL. Aliquots of 1 × 10^5^ cells were then seeded in triplicate into 96 well plates which were previously prepared with 90 µL/well of growth medium. After settling for 1 h, cells were stimulated with 500 ng/mL lipopolysaccharide (LPS from *E. coli* O111:B4, γ-irradiated, BioXtra, suitable for cell culture, L4391-1MG, Sigma-Aldrich Chemie GmbH) for 2 h and incubated at 37 °C and 5% CO_2_. At the end of the culture, the medium was transferred to another 96-well plate and frozen (−20 °C). The spent culture medium and the Tempus tubes containing blood samples were then shipped to the Royal Veterinary College (London, UK) frozen on dry ice for subsequent analysis.

### 2.4. Measurement of IL-1B and Nitric Oxide

Concentrations of IL-1B were measured in spent medium using an ELISA kit specific for boIL-1ß (Thermo Scientific, Rockford, IL, USA) as previously described [19]. The determination of nitric oxide (NO) concentration in medium was carried out using Griess reagents as previously described [19]. Briefly, a two-fold standard curve of 128 µM sodium nitrite in 2% FCS MØ media and sample supernatants were placed in a flat 96-well clear plate and mixed with equal volumes of Griess reagent (1:1 solutions A and B). After 10 min incubation, absorbance was analysed at 550 nm using a spectrophotometer (Spectramax M2, Molecular Devices, Wokingham, UK).

### 2.5. RNA Extraction

RNA was extracted from the whole blood samples using Tempus spin kits (Thermo Fisher Scientific, Hemel Hempstead, UK) following the supplied protocol. RNA quantity and integrity were assessed using an Agilent BioAnalyzer 2000 (Agilent, Milton Keynes, UK) and Agilent RNA 6000 Nano Kit (Agilent, UK). RNA measurements were also validated using a NanoDrop 1000 (Thermo Fischer Scientific, UK). All extracted RNA samples had a good integrity (RIN number >9.3) with concentrations between 40 and 163 ng/µL. They were kept at −80 °C for subsequent RNA sequencing. Quality control data are provided in Supplementary Table S2.

### 2.6. RNA-Sequencing, Mapping and Quantification

The extracted whole blood RNA samples were sent to Novogene Company Ltd. (Hong Kong, China) for RNA sequencing. After removing rRNA using a Globin-zero Gold rRNA removal kit (Illumina, San Diego, CA, USA), 400 ng total RNA was used for the preparation of RNA-Seq libraries with 250–300 bp insert strand specific library. The cDNA libraries were pooled and sequenced on Illumina NovaSeq platform with paired-end 150 bp sequencing (PE150) to reach over 30 million reads per sample. RNA-Seq analysis was carried out using a CLC Genomics Workbench V21 (Qiagen Digital Insights, Redwood City, CA 94063, USA) and its built-in workflows for RNA-Seq analysis. The poor quality reads were trimmed and the reads which passed quality control were mapped to a reference genome of *Bos taurus* assembly (ARS-UCD1.2, provided by GenBank). The gene expression (GE) values were quantified as reads per gene and reads per kilobase of transcript per million mapped reads (RPKM). These were stored as GE files in CLC Genomics Workbench for the analysis of differentially expressed genes (DEGs).

For variant analysis, reads were cleaned using Trimmomatic v. 0.36 (Trimmomatic: A flexible read trimming tool for Illumina NGS data. Available online: http://www.usadellab.org/cms/?page=trimmomatic. Accessed on 1 March 2021). The quality of raw and cleaned FASTQ files was assessed with FastQC (A quality control tool for high throughput sequence data. Available online: https://www.bioinformatics.babraham.ac.uk/projects/fastqc/. Accessed on 5 March 2021). *Bos taurus* assembly (ARS_UCD1.2), and its corresponding gene set was used as reference to map reads using the splice aware aligner HISAT2 [20]. Then, SAM files were converted to BAM files and coordinate sorted with SAMtools [21]. BAM files were further processed with Picard Tools (Picard. Available online: http://broadinstitute.github.io/picard/. Accessed on 15 March 2021) to mark PCR duplicates, add read group information, sort by chromosome and create indexes.

### 2.7. Analysis of Differentially Expression between Groups

DEGs between the groups were determined using an analysis of variance (ANOVA)-like model built in CLC Genomics Workbench V21. This included trimmed mean and Z-score normalisations across all samples and statistics based on a negative binomial generalised linear model. The cows were classified according to then lactation number as primiparous (PP, *n* = 6) or multiparous (MP, *n* = 9). The initial analysis showed that there were significant differences in leukocyte gene expression between the PP and MP cows. Therefore, the statistical model to examine the effect of *E. coli* mastitis on global gene expression of circulating leukocytes included mastitis group as a test variable and lactation group as confounding variable to control the differences of gene expression arising from number of lactations. Further information on group definitions is given in the Results section and Supplementary Table S1. Fold changes were derived from the RPKM values. The genes with an absolute fold change ≥1.5 in pairwise comparisons between the groups were selected for subsequent analysis. Benjamini–Hochberg (BH) procedure was used to adjust the *p*-values for multiple tests and significance was considered at *p* < 0.05.

### 2.8. Enrichment, Pathway and Cluster Analysis

Gene Ontology (GO) enrichment analysis was carried out using Partek Genomics Suite V7.1 (Partek Incorporation, Chesterfield, MO 63005, USA) with the genome version of ARS-UCD1.2 and the GO database formatted and updated by the software provider. The up- and down-regulated DEGs were separately analysed for GO enrichment with the focus on “Biological functions”. Fisher’s exact test with BH adjustment was used and statistical significance was considered at *p* (BH) < 0.05. The enrichment score (ES) was calculated as the negative natural logarithm of the enrichment *p*-value. The higher the enrichment score, the more over-represented this functional group was in the input gene list, with any ES >3 indicating significant enrichment. The DEG were also taken forward for pathway and cluster analysis using DAVID bioinformatics resources version 6.8 (The Database for Annotation, Visualization and Integrated Discovery (DAVID). Available online: https://david.ncifcrf.gov/. Accessed on 10 April 2022) [22,23] with *Bos taurus* as background. Fisher’s exact test with BH adjustment was used and statistical significance was considered at *p* (BH) < 0.05.

### 2.9. Variant Calling of Reads from RNA-Seq

Variant calling of the RNAseq data was performed following GATK best practice (GATK v3.8 [24]). Firstly, we used the GATK tool SplitNCigarReads to split reads into exon segments and hard-clip any sequences overhanging into the intronic regions; then, we used the Haplotype Caller to call variants in genomic blocks (gVCF mode) producing individual VCF files. A joint genotyping was performed on all gVCF files in order to create the variant call-set using GentoypeGBVFs tool. A series of hard allelic filters were applied as recommended by GATK germline best practices to prune low quality variants calls. The last step was to apply a hard filter (-window 35 -cluster 3 -filterName FS -filter “FS > 30.0” -filterName QD -filter “QD < 2.0”) to the joint file to optimise both high sensitivity and specificity. Variant calling was performed using the Ensembl VEP tool [25]. Linkage disequilibrium (LD) between autosomal variants up to 10 Mb apart across the genome extent was estimated in PLINK v1.9 [26] using the squared correlation between pairs of loci (r^2^) across autosomes and D’.

### 2.10. Statistical Analysis

The cow phenotype parameters (lactation number, yield, days in milk at sample collection) were expressed as mean ± SE. Differences between the groups were examined using a one-way ANOVA built in SPSS V28 (Chicago, IL, USA). The level for statistical significance was set at *p* < 0.05.

## 3. Results

### 3.1. Group Characteristics

The phenotype data from individual cows including their clinical results are given in Supplementary Table S1. One cow initially classified as *E. coli*(+) (A7) was omitted from the study as it had no clinical symptoms. This left nine cows per group which did or did not suffer from *E. coli* mastitis during the recruitment period, and all these animals were subsequently taken forward for the genetic variant analysis. All except one *E. coli*(+) cow were treated with antibiotics and NSAIDs as described above. Comparing the *E. coli*(+) and *E. coli*(−) control cows, their lactation numbers were 2.2 ± 0.5 and 3.0 ± 0.8, 305 day milk yields were 9835 ± 263 and 10,519 ± 205 kg and the days in milk at sample collection were 133 ± 18.5 and 112 ± 17.8 days, respectively (mean ± SEM). None of these measurements were significantly different.

Further sample selection was made prior to the RNAseq DEG analysis. Firstly, the *E. coli*(+) cows were divided into two subgroups based on the number of days from initial diagnosis until blood sample collection: these were EARLY (10.3 ± 1.8 days, range 6–17 days, *n* = 6) and LATE (46.7 ± 11 days, range 27–65 days, *n* = 3). Secondly, the three control cows with infected milk samples including the presence of Streptococci and Enterobacter (cows A6, A18 and A19, see Supplementary Table S1) were also excluded, leaving the control group (CONT) with *n* = 6 cows.

### 3.2. In Vitro PBMC Responses

Isolated PBMCs cultured in vitro and stimulated with LPS increased the production of IL-1ß as expected. There were no differences detected in the measurements of NO or IL-1ß produced by isolated PBMCs without LPS stimulation with respect to the *E. coli* status of the cows (Table 1). After LPS stimulation, there was a trend (*p* = 0.058) for the *E. coli*(+) LATE cows to produce a slightly higher concentration of NO than the CONT cows.

### 3.3. Differential Gene Expression between the Groups

**Figure 1:**
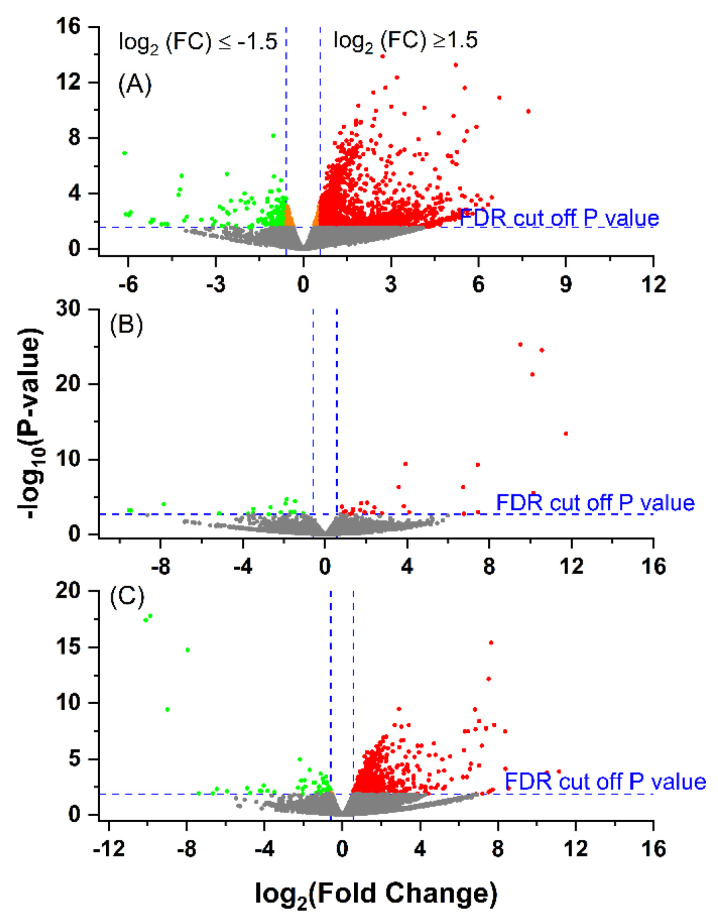
Volcano plot showing the gene expression profiles following an *E. coli* mastitis infection between: (**A**) EARLY (*n* = 6) and Control (CONT, *n* = 6); (**B**) LATE (*n* = 3) and CONT and (**C**) EARLY and LATE. The reads were quantified as reads per kilobase million (RPKM) and normalised with trimmed mean and Z-score across all samples. The fold changes were log2-tramsformed. The *p*-values were transformed with −log10. Cut-off point was *p* < 0.05 and absolute fold change ≥1.5. The cut-off *p* (raw) value for false discovery rate control (FDR) at *p* < 0.05 was 0.0275 for EARLY vs. CONT, 0.0019 for LATE vs. CONT and 0.0134 for EARLY vs. LATE. The green dots indicate significantly down-regulated genes; the red dots indicate significantly up-regulated genes; and the orange dots are the genes with FDR *p* < 0.05, but with absolute fold changes <1.5.

**Figure 2:**
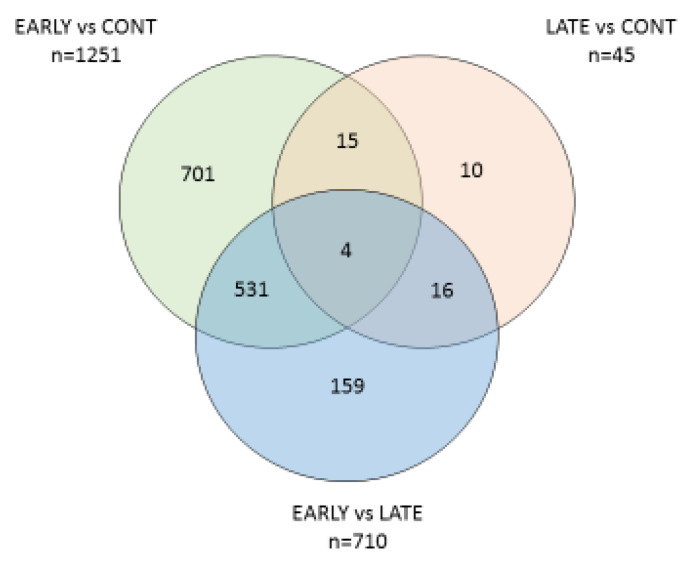
Venn diagram showing the numbers of DEGs identified between the three groups of cows.

After mapping sequencing reads to the *Bos taurus* reference genome (ARS-UCD 1.2), 20,110 out of 35,158 genes/transcripts were represented. The three groups of *E. coli*(+) (EARLY), *E. coli*(+) (LATE) and *E. coli*(−) (CONT) were then compared. Volcano plots showing the differential expression between groups is given in Figure 1. The numbers of DEG for each condition and those shared between them are illustrated in a Venn diagram (Figure 2) and the full gene lists are given in Supplementary Table S3A–C. The Venn diagram shows that there were 1251 DEGs between the CONT and EARLY cows but only 45 DEGs between CONT and LATE. For the two groups of *E. coli*(+) cows, there were 710 DEGs between the EARLY and LATE animals. The majority of these (535/710, 75%) were found in common between the CONT vs. EARLY and the EARLY vs. LATE comparisons. This indicates that the EARLY cows, sampled at 6–17 days after diagnosis, showed a very different leucocyte expression profile to the CONT animals but this was no longer the case in the LATE cows, sampled 27–65 days after diagnosis.

### 3.4. Comparison between E. coli(+) (EARLY) and E. coli(−) (CONT) Cows

In the comparison between the CONT and EARLY cows, there were 1090 up-regulated DEG in the *E. coli*(+) cows but only 161 were down-regulated (Supplementary Table S3A). Many of the top 20 up-regulated DEGs, with fold changes ranging from 38 to 209, were involved in a clear theme of immune functions and inflammation, including five antimicrobial peptides (AMP) (*CATHL1*, *CATHL2*, *CATHL4*, *CATHL5*, *CATHL6*) and three genes from the serpin family (*SERPINB4*, *LOC112445470*, *LOC511106*). Several top 20 genes encoded proteins with putative roles in cell motility and adhesion (*ACTA1*, *ACTBL2*, *CCN1*, *CDH13*, *MFAP5*, *PVALB*). The pro-inflammatory cytokines *IL1B*, *IL12B* and *IL18* were significantly up-regulated by 2.5–2.7-fold. A number of interleukin receptors (*IL1R1*, *IL1R2*, *IL1RL1*, *IL2RA*, *IL6R*, *IL13RA1*, *IL15RA*, *IL17RD*, *IL18R1*, *IL20RB*, *IL21R*), their associated proteins (*IL1RAP*, *IL18RAP*) and the interleukin 1 receptor antagonist (*IL1RN*) were also significantly up-regulated. In the TNF family, only *TNFSF14* (TNF superfamily member 14), *TNFAIP6* (TNF alpha induced protein 6) and *TNFRSF1A* (TNF receptor superfamily member 1A) were in the up-regulated list. The biological functions of the top 20 down-regulated DEGs were more diverse, and the majority encoded proteins whose functions are poorly characterised. Those with known immune activity included *C1R*, *LILRA4* and *LOC534578*. Three of the most highly down-regulated genes (*LOC505052*, *MGC151921,* and *LOC101904044*, with 29–63% reduction in expression) are predicted to encode odorant binding proteins.

### 3.5. Comparison between E. coli(+) (LATE) and E. coli(-) (CONT) Cows

The LATE cows were sampled on average 47 days after their initial diagnosis of *E. coli* mastitis and by this time, there was relatively little difference in their leucocyte gene expression in comparison with the CONT cows, with 29 up-regulated DEG and only 16 down-regulated (Supplementary Table S3B). Six of the most up-regulated genes encoded haemoglobin sub-units (*HBA1*, *HBB*, *HBE1*, *HBE4*, *HBG*, and *LOC528470*) while eight were predicted to encode for multidrug resistance-associated proteins (*LOC112449109*, *LOC509854*, *LOC107131247*, *LOC101902462*, *LOC107131271*, *LOC107131273*, and *LOC100848700*). The most down-regulated gene *LOC505052* encodes for an odorant binding protein and was also identified in the EARLY vs. CONT comparison. Four other highly down-regulated genes were predicted to encode for leukocyte immunoglobulin-like receptors (*LILRA4*, *LOC100852090*, *LOC790181*, and *LOC790255*).

### 3.6. Comparison between E. coli(+) (EARLY) and E. coli(+) (LATE) Cows

This comparison yielded 710 DEG, among which 662 were up-regulated and 48 down-regulated in the EARLY group cows (Supplementary Table S3C). The top 20 up-regulated genes again included four encoding cathelicidins and five serpins indicating that these were more highly expressed in the early period after infection. Of the other genes, *AZU1*, *KLRF2* and *PGLYRP1* also have antimicrobial activity while *MMP8*, *PLAT* and *PCOLCE2* have roles in remodelling extracellular matrix. Most of the down-regulated genes in this comparison (i.e., having a higher expression in the LATE cows) were the same as those which were up-regulated in the LATE vs. CONT comparison. The top 20 list included all six of the genes encoding haemoglobin subunits and three of the same genes encoding multidrug resistance-associated proteins. Of the DEG identified in the EARLY vs. LATE comparison, 535 were common with the EARLY vs. CONT comparison (Supplementary Table S3A). For all except four of these, the change was in the same direction, i.e., those genes which were up-regulated in the EARLY cows were more highly expressed than in either CONT or LATE cows. The exceptions were *DNER*, *LOC101902462*, *LOC112449072* and *LOC112449109*. These were all up-regulated in the EARLY vs. CONT comparison but down-regulated in EARLY vs. LATE. These encode the Delta/notch-like EGF-related receptor and three multidrug resistance-associated protein 4-like proteins.

### 3.7. GO Enrichment and Cluster Analysis for Gene Functions

The preliminary analysis suggested that the cows with *E. coli* mastitis were still exhibiting a high degree of inflammatory response around 10 days after their initial diagnosis and treatment. This response had largely ceased in the cows sampled between 4 and 9 weeks after diagnosis, but these animals nevertheless showed some longer-term changes in leukocyte gene expression. These two aspects were therefore taken forward for a more detailed investigation, mainly focusing on the genes which were up-regulated following infection.

**Figure 3:**
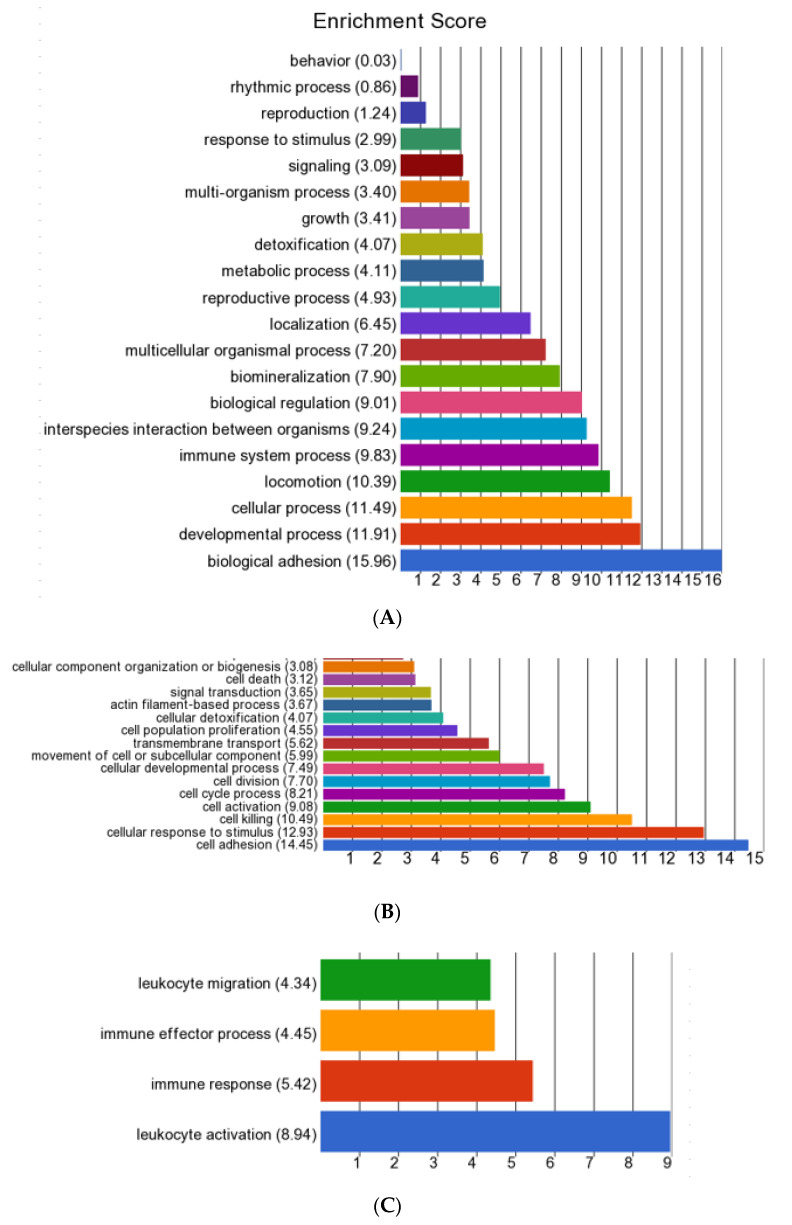
Summary of Gene Ontology (GO) enrichment analysis of the biological functions activated in the *E. coli*(+) EARLY cows in comparison with the *E. coli*(-) CONT cows (each *n* = 6). (**A**) All biological functions identified from the list of all up-regulated differentially expressed genes (DEGs, *n* = 1090); (**B**) sub-categories with an enrichment score (ES) >3 in the main pathway “Cellular process”; and (**C**) sub categories with an ES > 3 in the main pathway “Interspecies interaction”.

GO enrichment analysis of the up-regulated genes in the *E. coli*(+) EARLY cows vs. CONT identified 16 biological functions with an ES >3 (Figure 3, Supplementary Table S4A). Biological adhesion, containing 48 genes, had the highest ES of 15.96. Cell adhesion was also the top subcategory within cellular process (ES 11.49), in which the other top terms were the cellular response to stimulus, cell killing, cell activation and cell cycle process. There were 53 genes associated with locomotion (ES 10.39), in which the only two subcategories were taxis and cell motility. Within immune system process (ES 9.83, 66 genes) the top subcategory was leucocyte activation. In interspecies interaction between organisms (ES 9.24, 52 genes), the top subcategory was the killing of cells of other organisms. Genes involved in both adhesion and locomotion included *ADAM8*, *CD24*, *CD44*, *CDH13*, *CDK5R1*, *FN1*, *PARVA*, *RELN*, *S100A8*, *S100A9* and *SCARB1*. Those directly contributing to the killing of pathogens included those encoding antimicrobial peptides (*CATHL1*, *CATHL3*, *CATHL4*, *CATHL6*, *DEFB1*, *ELANE*, *HP*, *LTF*, *PGLYRP1*, *PGLYRP4*, *PTX3*, *S100A12*, *S100A8*, *S100A9* and *TREM1*), components of neutrophil azurophil granules (*AZU1* and *MPO*), macrophage activation (*ARG1*, *ARG2* and *TMEM229B*) and *SLC11A1*, an iron and magnesium cation transporter. Full gene lists relating to the identified subcategories are in Supplementary Table S4B.

GO enrichment analysis of the down-regulated genes in the EARLY vs. CONT comparison only yielded one significant biological function. This was an immune system process, with an ES of 12.79 and containing 20 DEG. These genes were mainly involved in antigen processing and presentation (*BLA-DQB*, *BOLA-DMA*, *BOLA-DOA*, *BOLA-DRA*, *BOLA-DRB3*, *CD40*, *CD79B*, *DSB*, *FCRL1*, *IFI30*) and leucocyte activation (*C1R*, *CD19*, *CXCL10*, *TLR10*, *TNFRSF21*).

The up-regulated DEG in the comparison between EARLY vs. CONT cows were also uploaded onto the DAVID Bioinformatics Resources website to generate functional annotation charts and clusters. The functional annotation chart displayed 89 functions and pathways significant with *p* (BH) < 0.05 and a fold enrichment >1.25. The full list is given in Supplementary Table S4C. The genes associated with the top terms are listed in Table 2. Functional annotation clustering identified 14 clusters with an ES > 2.5. These are listed in full in Supplementary Table S4D. These two approaches yielded similar results and are thus considered together. The main signalling pathways involved were bta04621:NOD-like receptor signalling pathway; bta04062: chemokine signalling pathway; bta05146: amoebiasis; and GO:0004908~interleukin-1 receptor activity. Pathway activation led to the increased expression of a number of genes encoding antimicrobial agents including six cathelicidins, four beta-defensins, three S100 calcium binding proteins, haptoglobin and lactoferrin. The genes identified under KEGG pathway bta1523-antifolate resistance included *FOLR3* (encoding folate receptor alpha), IL1B, four genes encoding ATP binding cassette subfamily members (*ABCA6*, *ABCA7*, *ABCA13* and *ABCB11*) and ten genes predicted to encode multidrug resistance-associated protein 4-like proteins, which are also members of the ABC superfamily.

Finally, the two lists of DEGs, which were more highly expressed in the LATE cows compared with either CONT (*n* = 29) or EARLY (*n* = 48) cows, were merged to generate a list of 62 DEGs (of which 15 were in common) which were more highly up-regulated in the leucocytes of cows in the LATE recovery phase from an *E. Coli* infection (Supplementary Table S3D). This list was uploaded into DAVID. Cluster analysis yielded four clusters with *p* (BH) < 0.05 and a fold enrichment >1.25 (Supplementary Table S4E). Cluster 1 included five genes encoding haemoglobin subunits; Cluster 2 contained five multidrug resistance-associated protein 4-like transporters; and Cluster 3 contained genes mainly involved in cholesterol biosynthesis. Cluster 4 contained a wider selection of 20 genes encoding proteins with at least one transmembrane domain. GO enrichment analysis and DAVID chart analysis revealed similar findings (data not shown).

### 3.8. Variant Calling

Variant calling from RNA-seq data can be unreliable due to missing genotypes arising from gaps in sequencing coverage. A stringent filtering process was therefore applied to avoid this issue. The initial output generated over 1.3 million variants. *E. coli(+)* and *E. coli*(−) cows had 828,016 variants in common spanning over 14,810 genes. A further 552,615 variants (overlapping 15,697 genes) were uniquely present in *E. coli*(+) cows and 514,179 (overlapping 15,008 genes) were found in *E. coli*(−) cows. Only variants found in both *E. coli*(+) and *E. coli(*−) cows were taken forward and further filtering was applied. Firstly, they were required to have a minor allele frequency (MAF) > 0.8. This implied that, in either the *E. coli*(+) or *E. coli*(−) group, over 80% of the cows had to have a variant differing from the allele most commonly present in the *Bos taurus* population. Secondly, the variant had to be present in ≥8 of the 9 cows in one group but in ≤1 of the 9 cows in the other group. This generated a list of 94 genetic variants found in 38 different genes which were almost fixed in either the *E. coli*(+) or *E. coli*(−) cows (Supplementary Table S5A).

Each variant was then linked to the corresponding transcript to investigate whether the variants influenced gene expression. This showed that 12 genes containing the identified variants were differentially expressed between *E. coli*(+) EARLY and CONT cows (Table 3). Among these, 11 were up-regulated in *E. coli*(+) cows with fold changes between 1.5 and 3.3. Just one gene, *BOLA-DOA*, had a 1.5-fold reduction in expression. Using assembly ARS-UCD1.2, three DEGs (*WIPI1*, *NC_037346.1* (*61752501..61782685*); *ARSG NC_037346.1* (*61781773..61886526*, *complement* and *SLC16A6, NC_037346.1 61867803..61877805*)) were in adjacent positions on BTA 19 (https://www.ncbi.nlm.nih.gov/gene/528410, accessed 26 April 2022) and these were found to be in linkage disequilibrium (r^2^ = 0.58 and D’ = 0.86).

More information on the variants identified in genes having differential expression between *E. coli*(+) and *E. coli*(−) cows is given in Supplementary Table S5B. The majority were intron variants, which can impact alternative splicing by interfering with splice site recognition. The variants in *WIPI1* and *BOLA-DOA* were located 3’ of the gene. All the variants had an impact classification of “Modifier”. This indicates that they may affect protein production, but predictions are difficult to make.

In addition, all 76 genes containing variants listed in Supplementary Table S5A were input as a gene list into a DAVID functional annotation analysis. Significance was not reached when an FDR correction was applied but this analysis did provide some indicative information, summarised in Table 4. Three genes (*DPYD*, *ACO2*, *NARFL*) are involved in iron–sulphur (Fe-S) clusters. A further four genes (*GSK3B*, *PPP3R1*, *RAC2* and *AKT1*) act at various points in the B-cell receptor signalling pathway.

## 4. Discussion

*E. coli* is a widespread environmental pathogen which is one of the major causes of clinical mastitis. Severe local effects are often multiplied by an aberrant immune response, and infection sometimes leads to the development of serious systemic symptoms [27]. Most previous studies using transcriptomic profiles to investigate the response to *E. coli* infections have used intra-mammary inoculation [6,15,16,17]. This approach has the advantage of being easy to control. The susceptibility to disease and its subsequent severity are, however, also influenced by the cow’s defence status [6] and genotype [4,28]. These aspects may be more closely reflected in naturally occurring cases compared with experimental models. The present study therefore compared the transcriptomic gene expression profiles in circulating leukocytes between cows with naturally occurring clinical mastitis caused by *E. coli* and paired samples from healthy cows on the same commercial farm.

These samples were necessarily collected at varying intervals of 6–65 days after the initial diagnosis. *E. coli* infections causing acute clinical mastitis are generally of short duration [11]. It was estimated that acute infections could usually be cleared within 5–12 days [29]. Blum et al. [30] divided the responses to *E. coli* into two stages. The acute phase, involving establishment of the infection, leucocyte infiltration and mammary gland inflammation started within a few hours, lasted for 1–2 weeks and was associated with local tissue damage. This was followed by a chronic resolution phase, during which milk parameters gradually recovered over several more weeks, although there might be a longer-term reduction in milk quality and yield [30]. Continued leukocyte infiltration into the mammary gland during this second phase involved both mononuclear cells and polymorphonuclear leukocytes, contributing to tissue repair processes. In support of this, the somatic cell count was previously shown to reduce to basal levels within 88 h of a mammary inflammation induced by LPS [18]. We therefore anticipated that the cows in our study, which received appropriate veterinary treatment following their diagnosis, would have largely recovered from the acute phase of infection at the time of blood sample collection. On the other hand, some investigators reported persistent cases of *E. coli* infection, in which bacteria were still recoverable after 40 days [29]. This is most likely due to longer survival within an intracellular reservoir in mammary epithelial cells, which could be associated with the strain of bacteria and/or to intrinsic differences in immune responses between cows [7,29]. The EARLY cows in the present study, sampled on average 10 days post infection, still showed clear evidence of a strong ongoing inflammatory response at this time. This does not necessarily imply that infective bacteria were still present, as the response could be associated with the removal of necrotic epithelial cells. This inflammation had, however, largely resolved in the LATE cows, sampled after at least 27 days had elapsed.

### 4.1. Evidence for an Ongoing Inflammatory Response during the Resolution Stage of an E. coli Infection

We identified 1090 up-regulated DEGs (but only 161 down-regulated DEGs) in leucocytes obtained from the *E. coli*(+) EARLY cows in comparison with CONT animals. The GO function and cluster analyses showed that the majority of up-regulated DEGs were involved in the activation of systemic immunity and inflammation. This was mainly achieved through the activation of the NOD-like receptor, chemokine and TLR4/IL1 signalling pathways. This closely agreed with previous studies on mammary gene expression using intramammary inoculation with *E. coli* which similarly reported the upregulation of the pathways of TLR signalling, NOD-like receptor (NLR) signalling, chemokine signalling and cytokine–cytokine interaction [16]. NLRs are a family of cytosolic pattern recognition receptors which detect specific PAMPs or host-derived damage signals (DAMPs) in the cytosol and can cooperate and interact with TLRs to regulate inflammatory processes such as NF-kappa B-/AP-1-dependent expression of pro-inflammatory cytokines and apoptosis [31]. In this study, genes encoding IL1 and IL18 receptors and the peptidoglycan recognition protein PGLYRP1 were all up-regulated. The latter stimulates TREM1 (triggering receptor expressed on myeloid cells). TREM1 also detects DAMPs and PAMPs and is a key activator of cytokine production, T-cell proliferation and activation of antigen-presenting cells [32]. Chemokines are an essential component of an inflammatory immune response to an infection by providing directional cues to recruit leukocytes to the site of inflammation. They also regulate many biological processes relating to the cellular activation, differentiation and survival of hematopoietic cells. Their role in recruiting leucocytes to the mammary gland following an *E. coli* mastitis infection has been recognised previously (e.g., [33,34]).

AMPs are multifunctional effector molecules which can kill pathogens through a variety of mechanisms [35]. Our recent study illustrated that the increased production of a variety of AMPs was related to the severity of mammary inflammation and was one of the main distinguishing differences in the way that circulating leukocytes responded to clinical compared with subclinical mastitis [36]. In the present study, *E. coli* infection induced the significant up-regulation of many AMPs including cathelicidins, beta defensins, S100A calcium binding proteins, lactoferrin and haptoglobin. Five cathelicidins were all in the top 20 up-regulated DEGs, with high fold changes. Cathelicidins and beta defensins are both families of small, cationic peptides which have a variety of bactericidal actions against both Gram-negative and Gram-positive bacteria [37]. Individual members of the S100 protein family are multifunctional proteins which readily form complexes including calprotectin, a heterodimer of S100A8 and S100A9. S100A proteins are implicated in regulating many intracellular and extracellular activities including cell differentiation, the stimulation of pro-inflammatory cytokines, induction of matrix metalloproteinases and the support of phagocytic properties through cytoskeletal rearrangement [38,39]. Both haptoglobin and lactoferrin are acute phase proteins which are well-established biomarkers of clinical mastitis, having direct bactericidal activity against *E. coli* [39,40]. SLC11A1 (NRAMP1) is another key protein in antibacterial defence via its role as an iron and magnesium cation transporter, which is crucial in iron homeostasis [41]. These various AMPs can act synergistically to clear infections by improving epithelial defences and prevent pathogen colonisation through both their direct antimicrobial actions and by fine-tuning other host immune responses and inflammation.

The KEGG pathway bta1523-Antifolate resistance was also up-regulated in the *E. coli*(+) EARLY cows. The genes identified included *FOLR3* (encoding folate receptor alpha), *IL1B*, four genes encoding ATP binding cassette subfamily members (*ABCA6*, *ABCA7*, *ABCA13* and *ABCB11*) and ten genes predicted to encode multidrug resistance-associated protein 4 (MRP4)-like proteins, which are also members of the ABC superfamily. These are low affinity, high-capacity ATP-driven transporters which move a wide variety of substrates including sugars, ions, amino acids, complex peptides, and hydrophobic (lipophilic) molecules across cell membranes [42] and they can therefore act as positive or negative regulators of metabolic pathways. Eight genes identified as encoding MRP4-like proteins were also among the top 20 most highly up-regulated genes in the *E.coli*(+) LATE cows. We also previously reported that the ABC transporter pathway was up-regulated in the leukocytes of a population of Holstein dairy cows with a low-energy balance status in early lactation [43]. These results suggest that ABC transporters are likely to play an important role in influencing leucocyte metabolic activity in response to increased ATP concentrations/consumption. However, the increase in these transporters could be a double-edged sword as ATP released by bacteria has recently been shown to limit IgA production as well as aiding the transport of nutrients, not only for the host cells but also for the bacteria [44,45]. The roles of different ABC superfamily members in response to mastitis therefore warrants further investigation.

The most up-regulated genes in the *E. coli*(+) EARLY cows also contained a number of DEGs predicted to encode for serpin-B3 and serpin-B4-like molecules (*SERPINB4*, *LOC511695*, *LOC786348*, *LOC112445470*, *LOC107131803* and *LOC511106*). We recently reported that three of these same genes were also amongst the most highly up-regulated in the same earlier study of cows with a low energy balance status, many of which were experiencing either mastitis or endometritis [43]. The serpin superfamily are serine/cysteine protease inhibitors which are strongly associated with a variety of inflammatory conditions, particularly through targeting areas with active thrombosis and/or thrombolysis [46,47]. The term GO:0002020~protease binding was also identified as up-regulated in *E. coli*(+) cows in the present study. Of the genes identified, *A2M* encodes alpha-2-macroglobulin which can act as an inhibitor of both thrombosis and fibrinolysis, involving the degradation of a fibrin clot by plasmin. These proteins are therefore likely to be playing a part in the response to vascular injury within the mammary glands of *E. coli* infected cows.

Far fewer genes (45 in total) were differentially expressed between the *E. coli* LATE and CONT cows, indicating that the cows had, by this time, largely recovered from their infection. The up-regulated list in the LATE cows mainly contained genes encoding haemoglobin subunits (*HBA1*, *HBB*, *HBE1*, *HBE4*, *HBG)* and multidrug resistance-associated proteins. In the present study, we used Tempus tubes and its isolation system. This extracts all the RNA present in blood and we did not separate the cell types. In addition to leukocytes, whole blood also contains reticulocytes. These are immature red blood cells derived from the bone marrow which usually circulate for about a day in the blood stream before developing into mature red blood corpuscles (RBCs). They account for 1–4% of the erythrocytes present in healthy adult humans [48]. Reticulocytes contain a network of ribosomal RNA which can be used for continued haemoglobin synthesis [49]. The increase in haemoglobin gene expression in the circulating blood of the LATE group of cows, several weeks after their initial *E. coli* infection, suggests an increase in the generation of new RBCs from bone marrow at this time. This may be needed to replace any damaged or lost RBCs during the earlier stages of infection.

### 4.2. Evidence for Genetic Differences between E. coli Infected and Healthy Cows

The likelihood of a particular cow developing clinical mastitis is mainly determined by the host defence status rather than by the pathogenicity of *E. coli* [6]. The bacteria need to overcome barriers of innate immunity to enter and proliferate in the mammary grand [50]. Many factors may contribute to the impairment of immune function and Rainard et al. [18] found marked differences between cows in their responses to low-dose LPS infusion. Previous evidence has indicated that genetic differences between cows results in varying susceptibility to infection [4,28]. Macrophages are key players in the body’s defence, having both pro-inflammatory and inflammation resolution activity [51,52,53]. Following the activation of the inflammasome complex, reactive oxygen species (ROS) are produced via a series of oxidative reactions [54,55]. The enzyme inducible nitric oxide synthase (iNOS) is also up-regulated, which then interacts with NADPH and ROS intermediaries to generate reactive nitrogen species (RNS) [56]. The bactericidal activity of macrophages mainly involves this interlinked production of ROS and RNS causing damage to cells and DNA [57,58]. Our previous work has shown that macrophages from Brown Swiss cattle produce significantly more RNS and less IL-1B in response to bacterial stimuli when compared to those from Holstein Friesian cattle and they also exhibited more efficient phagocytic activity and bacterial killing [19]. The present study therefore compared the basal and LPS-stimulated production of IL-1B and NO between the macrophages obtained from Holstein cows which did or did not succumb to an *E. coli* infection. LPS stimulated NO concentrations were slightly higher in the *E. coli*(+) LATE cows but there was no difference between the *E. coli*(+) EARLY and CONT groups. This study therefore did not provide any evidence that monocytes from cows of the same breed which did not catch an *E. coli* infection had a higher NO production capacity.

The clinical mastitis cases used in the present study occurred naturally and were paired in the analysis with samples collected from healthy cows. This enabled us to compare the prevalence of variants in genes expressed in leukocytes between cows which did or did not succumb to an *E. coli* infection when was exposed to the same environment. From the stringent analysis used, we identified 94 variants which were consistently present in at least 8 out of 9 cows in either the *E. coli*(+) or *E. coli*(−) groups but were rarely present or absent in the other group. The genes containing 12 of these variants were then found to be differentially expressed in leucocytes between the *E. coli*(+) EARLY and CONT cows. Among these, three DEGs (*WIPI1*, *ARSG* and *SLC16A6*) located together on BTA 19 came from a genomic region with an extended run of homozygosity (ROH) which was detected in a population of Shanghai Holstein cattle [59]. Analyses of ROH allow the identification of genomic regions with possible selection signatures for the breed, as their size and frequency vary according to population diversity and selection pressure. *WIPI1* encodes a member of the WD40 repeat family, and has been implicated in nucleophagy in differentiating keratinocytes during epidermal differentiation, with deficiency associated with skin disease [60]. This could potentially be important in maintaining good teat health. *ARSG* encodes a lysosomal sulfatase which hydrolyses sulphate esters and is involved in hormone biosynthesis, the modulation of cell signalling, and degradation of macromolecules. *SLC16A6* encodes a protein which transports lactate, pyruvate and beta-hydroxybutyrate across the cell membrane and is required for the hepatocyte secretion of ketone bodies during fasting [61]. These variants were in LD, and it remains to be determined which, if any, of these proteins may be relevant in the context of *E. coli* susceptibility.

Of the other variants identified, *RAC2* encodes a Rho-family GTPase that contributes to the B cell receptor signalling pathway and is involved in the phagocytosis of microbes and oxidative burst microbial killing. Mutations in this gene are associated with neutrophil immunodeficiency syndrome in humans [62]. Another SNP was present in *ARHGAP26*, which encodes Rho GTPase activating protein 26. This protein binds to focal adhesion kinase and mediates the activity of the GTP binding proteins RhoA and Cdc42 [63]. It was recently identified as a candidate gene for clinical mastitis in Sahiwal cattle, a *Bos indicus* breed [64].

Of the other variants in genes which showed an up-regulated differential expression between *E. coli*(+) and *E. coli*(−) cows, *EBF1* and *EYA3* are both involved in regulation of gene transcription. *GNG7* encodes guanine nucleotide-binding protein γ−7, whose expression level was associated with the infiltration of multiple immune cells into human colorectal cancer samples [65]. Little information is available concerning *FAM129A* (also called *NIBAN1*), although expression is up-regulated as part of the integrated stress response [66]. *PFKFB4* encodes an enzyme which regulates the concentration of the glycolytic by-product fructose-2,6-bisphosphate (F2,6BP) and there is evidence that it is important for the stimulation of glycolysis and activation of T-cells [67]. *CCND3* encodes cyclin D3, which contributes to the cell cycle G1/S-phase transition and is a key regulator of B-cell proliferation [68]. A further four genes containing variants (*GSK3B*, *PPP3R1*, *RAC2* and *AKT1*), but which were not differentially expressed, also act at various points in the B-cell receptor signalling pathway. Three further genes (*DPYD*, *ACO2*, *NARF1*) are involved in iron–sulphur (Fe-S) clusters. These act as small metallocofactors which are used to perform complex chemical reactions within several important cellular pathways, including redox catalysis, fatty acid oxidation and DNA repair and replication [69].

The highly polymorphic major histocompatibility complex (MHC) has been implicated in the resistance and susceptibility to a broad range of diseases including mastitis [70]. *BOLA-DOA* encodes the major histocompatibility complex, class II, DO alpha. This was the only variant identified in a gene with significantly lower expression in the *E. coli*(-) cows. Class II molecules are expressed on antigen-presenting cells and are important for driving T-cell development and differentiation. The bovine MHC region of BTA23 has been under significant selection pressure during the development of the Holstein breed, with significantly decreased heterozygosity in contemporary animals [71]. A high proportion of the relatively few genes which were down-regulated in *E. coli* infected cows were enriched in those encoding proteins important for antigen processing and presentation in both the EARLY (*BLA-DQB*, *BOLA-DMA*, *BOLA-DOA*, *BOLA-DRA*, *BOLA-DRB3*) and LATE cows (four genes encoding leukocyte immunoglobulin-like receptor subfamily A members). The latter are widely expressed throughout the body and interact with collagen and MHC class 1 molecules. They may influence the signalling pathways of both the innate and adaptive immune systems [72] and were implicated in causing an immunosuppressive environment [73]. Together, these results therefore support earlier work in suggesting that cows which are more susceptible to *E. coli* mastitis have potentially important alterations in their MHC system.

### 4.3. Study Limitations

This was an observational study performed on a commercial farm, which meant that some aspects of the investigation could not be as well controlled as would be possible at a research establishment. Firstly, all cows diagnosed with *E. coli* mastitis were treated as deemed most appropriate by the attending veterinarian, with most receiving both NSAID and antibiotic therapy. The inflammatory response observed in blood samples obtained at the follow-up visit some days later would presumably have been even more pronounced in the absence of treatment. This should not, however, have influenced the comparison of the genetic variants between cases and control cows. Secondly, we did not assess whether there were still viable bacteria present in the milk at the time of blood sample collection and thirdly the timing from diagnosis to blood sample collection varied from 6 to 17 days for the *E. coli*(+) EARLY cows. We therefore could not know how quickly individual animals recovered from infection. Nevertheless, the EARLY cows were still clearly undergoing an inflammatory response. This could have been associated with continuing clearance of necrotic tissue from their mammary gland. There is, however, evidence of *E. coli* survival in the mammary gland for up to at least 40 days in some cows which develop a persistent infection [7,29]. While speculative, this situation would possibly be more likely to occur in commercial animals experiencing natural infections in comparison with studies using challenge models performed on previously healthy cows kept under research conditions.

## 5. Conclusions

This study found that leukocytes from *E. coli*-infected cows showed an up-regulation of NOD-like, interleukin-1 receptor and chemokine signalling pathways resulting in the increased expression of genes encoding a broad spectrum of antimicrobial peptides. This provided a clear indication that the cows were still actively engaged in immune and inflammatory responses to infection in their second week post diagnosis, despite having received standard veterinary treatment. Inflammation had, however, largely resolved within four weeks. A search for genetic variants between cows which did or did not become infected with *E. coli* during the same period identified 94 genetic variants in 38 different genes, of which 12 showed a differential expression between control and infected cows. These findings supported existing evidence that mastitis susceptibility is influenced by genes encoding Rho-family GTPases and the major histocompatibility complex, affecting antigen presentation and processing. A better understanding of how cows respond to infection is essential for both short-term improvements in treatment options and the longer- term goal of breeding more disease-resistant cows.

## Figures and Tables

**Table 1:** Production of NO and IL1B in vitro by PBMCs isolated from *E. coli*(−) and *E.* *coli*(+) cows with or without stimulation with 500 ng/mL LPS for 2 h.

	*E. coli*(−) * CONT	*E. coli*(+) EARLY ^‡^	*E. coli*(+) LATE ^‡^
*n*	6	6	3
Control NO production (µM)	8.7 ± 0.37	10.3 ± 1.72	8.6 ± 0.06
LPS stimulated NO production (µM)	8.5 ± 0.18 ^#^	8.5 ± 0.13	9.4 ± 0.56 ^#^
Control IL1B production (pg/mL)	87.0 ± 18.58 ^a^	59.8 ±11.87 ^a^	61.3 ± 14.83 ^a^
LPS stimulated IL1B production (pg/mL)	258.3 ± 86.90 ^b^	195.5 ± 40.08 ^b^	205.7 ± 38.84 ^b^

* The 3 control cows which had evidence of subclinical mastitis were omitted from the analysis. ^‡^ EARLY cows were sampled at 10 ± 1.8 days after diagnosis of clinical mastitis and LATE cows at 47 ± 11.0 days. Values are mean ± SEM. Within columns, b > a, *p* < 0.01. Within rows, ^#^ indicates a trend towards difference, *p* = 0.058.

**Table 2:** Gene lists from the main terms identified by DAVID chart analysis associated with differentially up-regulated genes in leukocytes collected from cows with *E. coli*(+) (EARLY) compared with healthy CONT cows (*n* = 6 cows/group) ^#^.

Category	DEGs
NOD-like receptor signalling pathway	*BCL2L1*, *CAMP*, *CATHL1*, *CATHL2*, *CATHL3*, *CATHL4*, *CATHL5*, *CATHL6*, *CXCL2*, *DEFB1*, *DEFB10*, *GABARAP*, *GABARAPL1*, *IFNAR1*, *IFNAR2*, *IL1B*, *IL18*, *LOC100301263*, *LOC112441458*, *LOC112443481*, *MAP1LC3A*, *MAPK13*, *MAPK14*, *MAPK3*, *MEFV*, *MYD88*, *NAIP*, *NFKBIA*, *NLRX1*, *PLCB1*, *STAT2*, *TXNIP*
Amoebobiasis	*ACTN1*, *ARG1*, *ARG2*, *CASP3*, *COL1A1*, *COL1A2*, *COL3A1*, *COL4A2*, *COL4A2*, *CXCL2*, *FN1*, *IL1B*, *IL1R1*, *IL1R2*, *IL12B*, *LAMB1*, *LAMC1*, *LAMC2*, *LOC505658*, *LOC511106*, *LOC786348*, *PIK3CD*, *PLCB1*, *RAB5C*, *SERPINB4*
Chemokine signalling pathway	*ARRB2*, *CCL16*, *CCR1*, *CCR6*, *CXCL13*, *CXCL2*, *CXCR1*, *CXCR2*, *CXCR4*, *FGR*, *FOXO3*, *GNG2*, *GNG7*, *JAK2*, *LOC100297044*, *MAPK3*, *NCF1*, *NFKBIA*, *PAK1*, *PARD3*, *PIK3CD*, *PIK3CG*, *PLCB1*, *PREX1*, *PTK2B*, *PXN*, *RAC2*, *STAT2*, *STAT3*, *TIAM1*, *XCR1*
Calcium	*ACTN1*, *ADGRE5*, *ALOX15*, *ALPL*, *ANXA1*, *ANXA9*, *CAPN1*, *CAPN3*, *CDH13*, *COL1A1*, *COL1A2*, *CPNE2*, *DYSF*, *EHD1*, *ENTPD1*, *F5*, *FBN1*, *FGG*, *ITGA3*, *LOXL2*, *MMP2*, *MMP8*, *MMP9*, *NOTCH2*, *PADI3*, *PADI4*, *PLA2G4A*, *PLA2G4F*, *PLCB1*, *PLCD1*, *PRSS2*, *PVALB*, *RASGRP4*, *RELN*, *RPH3A*, *RYR1*, *S100A12*, *S100A8*, *S100A9*, *SELL*, *SLC24A3*, *SPARC*, *SVIL*, *TGM1*, *TGM2*, *TGM3*, *TKT*, *TRPC5*, *TRPC6*, *TYROBP*
Interleukin receptors	*IL1R1*, *IL1R2*, *IL1RAP*, *IL1RL1*, *IL18R1*, *IL18RAP*, *MYD88*, *TGM2*
Cathelicidins and other antimicrobials	*CAMP*, *CATHL1*, *CATHL2*, *CATHL3*, *CATHL4*, *CATHL5*, *CATHL6*, *CHI3L1*, *COL1A1*, *COL1A2*, *CXCL13*, *CXCL2*, *DEFB1*, *DEFB10*, *DEFB4A*, *DEFB7*, *DPT*, *FN1*, *HP*, *LTF*, *PGLYRP1*, *PGLYRP4*, *PTAFR*, *S100A8*, *S100A9*, *S100A12*, *SCARB1*
Peptide cross-linking	*ANXA1*, *COL3A1*, *DSP*, *EPB42*, *FN1*, *TGM1*, *TGM2*, *TGM3*
Wound healing	*ALOX15*, *AQP1*, *CNN2*, *COL3A1*, *DSP*, *FN1*, *NOTCH2*, *PAK1*, *PARD*, *PTK7*, *SDC1*, *SLC11A1*, *YAP1*
Protease binding	*A2M*, *ANXA9*, *ATP9A*, *CDK5R1*, *COL1A1*, *COL1A2*, *COL3A1*, *ELANE*, *FLOT1*, *FN1*, *IL1R1*, *ITGA3*, *LOC506828*, *SELL*
Antifolate resistance and ABC transporters	*ABCA6*, *ABCA7*, *ABCB11*, *ABCA13*, *FOLR3*, *IL1B*, *LOC509854*, *LOC520016*, *LOC522174*, *LOC100337053*, *LOC100847574*, *LOC107131218*, *LOC107131247*, *LOC107131259*, *LOC107131271*, *LOC107131273*
Collagen	*COL1A1*, *COL1A2*, *COL3A1*, *COL4A2*, *COL6A1*, *COL6A2*, *COL6A3*, *CTHRC1*, *MMP2*, *PCOLCE2*, *PLOD3*

^#^ See Supplementary Table S4C for full list of terms. Gene lists from some related terms were merged.

**Table 3:** Genes with variants present in at least 8 out of 9 samples of *E. coli*(+) or *E. coli*(−) cows which were also differentially expressed between the EARLY and CONT groups of cows.

Gene Symbol	Max Group Mean	Fold Change	*p* (BH)	Group ^#^	BTA	Gene Position
*EYA3*	2.616	1.511	0.025	A	2	NC_037329.1 (125201889..125390272)
*RAC2*	6.971	1.871	0.000	A	5	NC_037332.1 (75656456..75673385)
*GNG7*	3.301	1.875	0.001	A	7	NC_037334.1 (21002517..21097631)
*ARHGAP26*	4.428	1.539	0.005	B	7	NC_037334.1 (53811496..54284856)
EBF1 *	2.895	−1.563	0.017	A/B	7	NC_037334.1 (70284253..70694732
*FAM129A*	31.780	2.061	0.000	A	16	NC_037343.1 (65828044..66012042,
*WIPI1*	2.243	3.271	0.000	A	19	NC_037346.1 (61752501..61782685)
ARSG *	1.651	1.929	0.008	A/B	19	NC_037346.1 (61781773..61886526)
*SLC16A6*	19.480	1.671	0.010	B	19	NC_037346.1 (61867803..61877805)
*PFKFB4*	9.899	1.883	0.001	B	22	NC_037349.1 (51321977..51363429)
*BOLA-DOA*	23.840	−1.539	0.006	A	23	NC_037350.1 (7314757..7323452)
*CCND3*	29.144	1.594	0.014	B	23	NC_037350.1 (15698650..15793268

^#^ SNP were identified on the basis of a minor allele frequency (MAF) of: (A) MAF > 0.8 in *E. coli*(−) cows and MAF < 0.3 in *E. coli*(+) cows; (B) MAF > 0.8 in *E. coli*(+) cows and MAF < 0.3 in *E. coli*(−) cows. * Genes with SNP identified in both *E. coli*(+) and *E. coli*(−) cows.

**Table 4:** DAVID functional annotation analysis of 38 genes identified containing variants which differed between *E. coli*(+) and *E. coli*(−) cows, showing the top 6 terms.

Category	Term	Genes	Fold Enrichment	*p*-Value	FDR
KEGG_PATHWAY	bta04310:Wnt signalling pathway	*GSK3B, TCF7L2*, *CAMK2D*, *PPP3R1*, *CCND3*, *RAC2*	8.06	0.0007	0.113
UP_KEYWORDS	4Fe-4S	*DPYD*, *ACO2*, *NARFL*	34.25	0.0033	0.320
KEGG_PATHWAY	bta05200:Pathways in cancer	*GSK3B*, *TCF7L2*, *DAPK1*, *GNG7*, *RASSF5*, *TPR*, *RAC2*, *AKT1*	3.70	0.0044	0.230
KEGG_PATHWAY	bta05210:Colorectal cancer	*GSK3B*, *TCF7L2*, *RAC2*, *AKT1*	11.16	0.0050	0.230
KEGG_PATHWAY	bta04662:B cell receptor signalling pathway	*GSK3B*, *PPP3R1*, *RAC2*, *AKT1*	10.52	0.0059	0.230
GOTERM_CC_DIRECT	GO:0005925~focal adhesion	*CCND3*, *PPP1R12A*, *RPLP0*, *RDX*, *RAC2*, *MYH9*	4.94	0.0067	0.391

## Data Availability

The RNA-seq fastq data can be obtained from NCBI Sequence Read Archive (https://submit.ncbi.nlm.nih.gov/about/sra/, accessed on 8 April 2022) BioProject PRJNA837869 with BioSample Accession number SAMN28232994.

## Supplementary Materials

The following supporting information can be downloaded at: https://www.mdpi.com/article/10.3390/ani12162146/s1, Supplementary Table S1.: Animal Information; Supplementary Table S2.: Concentration and quality of leukocyte RAN samples analysed with Agilent BioAnalyzer and NanoDrop; Supplementary Table S3A–D.: (A) DEG in comparison of *E. coli*(+) EARLY vs. CONT cows; (B) DEG in comparison of *E. coli*(+) LATE (*n* = 3) vs. CONT cows; (C) DEG in comparison of *E. coli*(+) EARLY vs. *E. coli*(+) LATE cows; (D) Combined list of up-regulated genes in *E. coli*(+) LATE cows (*n* = 3) versus either CONT or *E. coli*(+) EARLY; Supplementary Table S4A–E.: (A) GO enrichment analysis of the up-regulated genes in the *E. coli*(+) EARLY cows vs. *E.coli*(−) CONT cows, each *n* = 6; (B) Full gene lists relating to the subcategories identified from the GO enrichment analysis illustrated in Figure 3 of the up-regulated genes in the *E. coli*(+) EARLY cows vs. *E.coli*(−) CONT cows, each *n* = 6; (C) DAVID Bioinformatic Analysis. Functional annotation chart of up-regulated genes in comparison with *E. coli*(+)EARLY vs. CONT cows, each *n* = 6 cows; (D) DAVID Bioinformatic Analysis. Functional annotation clustering of up-regulated genes in comparison of *E. coli*(+) EARLY vs. CONT cows, each *n* = 6 cows; (E) Functional annotation clustering in comparison of all up-regulated genes which showed significant differential expression in leukocytes in the cows with *E. coli*(+) LATE compared with either the healthy CONT cows or the *E. coli*(+) EARLY group; Supplementary Table S5.: Common genetic variants between *E. coli* +ve and *E. coli* -ve cows; Supplementary Table S6: Annotation of variants identified in genes having differential expression between *E. coli*(+) and *E. coli*(−).
