# Supplementary material for: Transcriptomic Analysis of Circulating Leukocytes Obtained during the Recovery from Clinical Mastitis Caused by Escherichia coli in Holstein Dairy Cows
Source: Animals (Basel). 2022 Aug 21;12(16):2146. doi: 10.3390/ani12162146 (PMC9404729; doi:10.3390/ani12162146)
Supplement: Supplementary file 1 [file animals-12-02146-s001.zip › Supplementary Table S1 Animal Information.pdf]

Table S1. Animal Information.

Experiment ID	Lactation No.	305-d Milk yield (kg)	DIM	Interval (d) from *E. coli* diagnosis to sampling	Treated for *E. coli* infection	Udder Health (if not *E. coli*)	Group for analysis
A4	4	10401	106	6	YES	NA	*E. coli*(+) EARLY
A2	1	8972	133	8	NO	NA	*E. coli*(+) EARLY
A9	1	9649	149	8	YES	NA	*E. coli*(+) EARLY
A10	1	8735	139	8	YES	NA	*E. coli*(+) EARLY
A8	2	9247	96	15	YES	NA	*E. coli*(+) EARLY
A3	1	9745	19	17	YES	NA	*E. coli*(+) EARLY
A1	4	10211	205	27	YES	NA	*E. coli*(+) LATE
A5	4	11230	155	48	YES	NA	*E. coli*(+) LATE
A17	2	10322	193	65	YES	NA	*E. coli*(+) LATE
A7#	1	8746	39	44	NO	NA	
A11	1	10277	116	NA	NA	No germ history	*E. coli*(-) CONT
A12	1	9840	201	NA	NA	No germ history	*E. coli*(-) CONT
A13	2	10876	88	NA	NA	No germ history	*E. coli*(-) CONT
A14	2	9967	59	NA	NA	No germ history	*E. coli*(-) CONT
A15	3	10675	79	NA	NA	No germ history	*E. coli*(-) CONT
A16	3	11335	89	NA	NA	Missing	*E. coli*(-) CONT
A6‡	2	9872	93	NA	NA	Unspecific Streptococci, Unspecific Enterobacter, Unspecific germs	*E. coli*(-) CONT‡
A18‡	4	11488	81	NA	NA	Unspecific germs	*E. coli*(-) CONT‡
A19‡	9	10341	205	NA	NA	Unspecific Streptococci, Unspecific Enterobacter, Unspecific germs	*E. coli*(-) CONT‡

DIM, days in milk; NA, not aplicable; # Cow A7 omitted from all analyses as it had no clinical symptoms; ‡ Cows A6, A18 and A19 omitted from RNAseq analysis due to presence of udder infections other than *E. coli*.
