# Supplementary material for: Transcriptomic Analysis of Circulating Leukocytes Obtained during the Recovery from Clinical Mastitis Caused by Escherichia coli in Holstein Dairy Cows
Source: Animals (Basel). 2022 Aug 21;12(16):2146. doi: 10.3390/ani12162146 (PMC9404729; doi:10.3390/ani12162146)
Supplement: Supplementary file 1 [file animals-12-02146-s001.zip › Supplementary Table S5.pdf]

Supplementary Table S5. Common genetic variants between *E. coli* +ve and *E. coli* -ve cows. Only variants with a minor allele frequency (MAF) > 0.8 were included. This implied that, in either the *E. coli(+)* or *E. coli(-)* group, over 80% of the cows had to have a variant differing from the allele most commonly present in the *Bos taurus* population. Secondly, the variant had to be present in ≥8 of the 9 cows in one group but in ≤1 of the 9 cows in the other group. (A) Ranked based on MAF > 0.8 in *E. coli*(-) cows (56 variants); (B) Ranked based on MAF > 0.8 in *E. coli*(+) cows (38 variants). Asterisks indicate genes present in both groups A and B.

A

CHR	SNP	MAF pos	REF	ALT	No pos_ID	MAF neg	No neg_ID	Gene
NC_037330.1	9582229	0.056	T	G	9	0.944	9	*IGSF8**
NC_037332.1	68183061	0.056	T	A	9	0.944	9	*CHST11**
NC_037336.1	31758597	0.056	G	A	9	0.944	9	*MAN1A1*
NC_037334.1	70585950	0.063	G	T	8	0.833	9	*EBF1**
NC_037343.1	4352208	0.063	T	A	8	0.889	9	*RASSF5*
NC_037350.1	7314809	0.063	T	G	8	0.889	9	*BOLA-DOA*
NC_037343.1	67418274	0.063	A	G	8	0.944	9	*TPR**
NC_037343.1	67431369	0.063	C	G	8	0.944	9	*TPR**
NC_037348.1	69243133	0.063	G	T	8	0.944	9	*AKT1**
NC_037330.1	16028613	0.063	C	G	8	1	9	*UBE2Q1*
NC_037333.1	58212143	0.111	C	A	9	0.813	8	*TMEM156**
NC_037332.1	98033207	0.111	T	A	9	0.938	8	*ETV6**
NC_037332.1	116848067	0.125	A	G	8	0.833	9	*GRAMD4*
NC_037337.1	87461439	0.125	A	T	8	0.938	8	*GPATCH2L*
NC_037329.1	120195498	0.167	A	G	9	0.833	9	*EIF4E2*
NC_037331.1	46650427	0.188	G	*	8	0.889	9	*RINT1*
NC_037337.1	64817860	0.188	A	G	8	0.944	9	*SQOR*
NC_037342.1	20284619	0.222	T	A	9	0.813	8	*RDX*
NC_037329.1	120204692	0.222	C	G	9	0.833	9	*EIF4E2*
NC_037339.1	48775901	0.222	C	T	9	0.833	9	*KLF12*
NC_037330.1	105902539	0.222	G	C	9	0.875	8	*RLF*
NC_037334.1	66114681	0.222	C	T	9	0.875	8	*MRPL22*
NC_037335.1	83038346	0.222	T	C	9	0.875	8	*SLC35D2*
NC_037337.1	92496081	0.222	C	T	9	0.889	9	*GTF2A1*
NC_037346.1	63148591	0.222	C	T	9	0.944	9	*HELZ*
NC_037328.1	111780525	0.25	G	A	8	0.813	8	*GMPS*
NC_037329.1	23544011	0.25	C	G	8	0.813	8	*MAP3K20*
NC_037329.1	135215532	0.25	T	C	8	0.813	8	*RCC2*
NC_037330.1	2063880	0.25	G	A	8	0.813	8	*POGK*
NC_037334.1	21014556	0.25	G	C	8	0.813	8	*GNG7*
NC_037346.1	7670916	0.25	A	G	8	0.813	8	*DGKE*
NC_037353.1	33570520	0.25	G	T	8	0.813	8	*TCF7L2*
NC_037329.1	125367162	0.25	G	T	8	0.833	9	*EYA3*
NC_037337.1	92483283	0.25	C	T	8	0.833	9	*GTF2A1*
NC_037344.1	65492804	0.25	T	C	8	0.833	9	*GRK3*
NC_037328.1	64824563	0.25	T	C	8	0.875	8	*GSK3B*
NC_037330.1	105957688	0.25	A	C	8	0.875	8	*RLF*
NC_037331.1	19246835	0.25	A	C	8	0.875	8	*PHF14*
NC_037332.1	73548133	0.25	C	T	8	0.875	8	*TOM1*
NC_037332.1	112619591	0.25	T	C	8	0.875	8	*POLR3H*
NC_037343.1	66006047	0.25	A	G	8	0.889	9	*FAM129A*
NC_037346.1	61782120	0.25	T	C	8	0.889	9	*WIPI1*
NC_037346.1	61782120	0.25	T	C	8	0.889	9	*ARSG**
NW_020190994.1	553435	0.25	A	T	8	0.938	8	*LOC521568*
NC_037346.1	63193439	0.25	C	T	8	0.944	9	*HELZ*
NC_037329.1	118618124	0.25	A	C	8	1	8	*CAB39*
NC_037330.1	105958999	0.278	C	G	9	0.813	8	*RLF*

NC_037332.1	119573540	0.278	A	C	9	0.813	8	*SELENOO*
NC_037333.1	12106029	0.278	T	C	9	0.813	8	*CAMK2D*
NC_037336.1	38967523	0.278	T	C	9	0.813	8	*REV3L*
NC_037338.1	75057697	0.278	C	G	9	0.813	8	*UBXN2A*
NC_037332.1	74754774	0.278	T	A	9	0.833	9	*MYH9*
NC_037332.1	75664664	0.278	G	T	9	0.833	9	*RAC2*
NC_037337.1	12778812	0.278	A	G	9	0.833	9	*MEGF11*
NC_037346.1	63190965	0.278	A	G	9	0.938	8	*HELZ*
NC_037332.1	112598940	0.278	T	C	9	1	9	*ACO2*

B

CHR	SNP	MAF pos	REF	ALT	No pos_ID	MAF neg	No neg_ID	Gene
NC_037330.1	9582229	0.889	T	C	9	0.056	9	*IGSF8**
NC_037332.1	68183061	0.944	T	C	9	0.056	9	*CHST11**
NC_037333.1	90332951	1	C	*	9	0.056	9	*RCHY1*
NC_037338.1	66633374	0.944	C	G	9	0.056	9	*PPP3R1*
NC_037338.1	99480186	1	C	T	9	0.056	9	*NUP188*
NC_037343.1	67418274	0.938	A	C	8	0.056	9	*TPR**
NC_037343.1	67431369	0.938	C	A	8	0.056	9	*TPR**
NC_037345.1	65565962	0.813	T	G	8	0.056	9	*ZNF274*
NC_037348.1	69243133	0.938	G	C	8	0.056	9	*AKT1**
NC_037349.1	1460473	0.813	G	A	8	0.056	9	*SEC61G*
NC_037350.1	15736092	1	T	C	8	0.056	9	*CCND3*
NC_037351.1	34511012	0.938	T	A	8	0.056	9	*MIB1*
NC_037332.1	98033207	0.889	T	C	9	0.063	8	*ETV6**
NC_037332.1	9325814	0.833	C	T	9	0.111	9	*PPP1R12A*
NC_037334.1	70585950	0.875	G	C	8	0.111	9	*EBF1**
NC_037335.1	80834582	0.813	C	T	8	0.111	9	*DAPK1*
NC_037344.1	62560799	0.875	T	A	8	0.111	9	*RPLP0*
NC_037334.1	17327616	1	G	T	8	0.125	8	*LOC112447353*
NC_037335.1	41692579	0.833	A	C	9	0.167	9	*PUM3*
NC_037346.1	61874326	0.813	C	A	8	0.167	9	*SLC16A6*
NC_037346.1	61874326	0.813	C	A	8	0.167	9	*ARSG**
NC_037328.1	52952052	0.833	T	G	9	0.188	8	*IFT57*
NC_037329.1	4770561	0.875	C	A	8	0.188	8	*WDR33*
NC_037333.1	58212143	0.889	C	G	9	0.188	8	*TMEM156**
NC_037330.1	45990080	0.938	C	T	8	0.222	9	*DPYD*
NC_037334.1	54231683	0.833	A	G	9	0.222	9	*ARHGAP26*
NC_037340.1	18000820	0.813	T	C	8	0.222	9	*APBB1IP*
NC_037340.1	18000848	0.813	CA	TA	8	0.222	9	*APBB1IP*
NC_037340.1	18000913	0.813	G	A	8	0.222	9	*APBB1IP*
NC_037349.1	51327126	0.938	A	G	8	0.222	9	*PFKFB4*
NC_037352.1	631269	0.813	T	C	8	0.222	9	*NARFL*
NC_037333.1	98245426	0.813	G	A	8	0.25	8	*ABRAXAS1*
NC_037330.1	6649228	0.833	G	T	9	0.278	9	*HSD17B7*
NC_037330.1	14874291	0.889	G	C	9	0.278	9	*SYT11*
NC_037334.1	14191941	0.944	A	C	9	0.278	9	*ZNF699*
NC_037338.1	100376121	0.813	C	G	8	0.278	9	*FNBP1*
NC_037340.1	12673908	0.813	C	T	8	0.278	9	*USP6NL*
NC_037344.1	16795936	0.813	G	A	8	0.278	9	*RNF150*
