# Supplementary material for: Transcriptomic Analysis of Circulating Leukocytes Obtained during the Recovery from Clinical Mastitis Caused by Escherichia coli in Holstein Dairy Cows
Source: Animals (Basel). 2022 Aug 21;12(16):2146. doi: 10.3390/ani12162146 (PMC9404729; doi:10.3390/ani12162146)
Supplement: Supplementary file 1 [file animals-12-02146-s001.zip › Supplementary Table S6.pdf]

Supplementary Table S6. Annotation of variants identified in genes having differential expression between *E. coli(+)* and E. coli(-).

Gene	BTA	SNP	MAF POS	REF	ALT	+ve cows (n)	MAF NEG	-ve cows (n)	Consequence	Impact	Gene_ensembl	Existing varn
EYA3	2	125367162	0.25	G	T	8	0.833	9	intron_variant	MODIFIER	ENSBTAG00000043989	rs110270588
RAC2	5	75664664	0.278	G	T	9	0.833	9	intron_variant	MODIFIER	ENSBTAG00000011043	rs137621320
GNG7	7	21014556	0.25	G	C	8	0.813	8	intron_variant	MODIFIER	ENSBTAG00000007644	rs209187815
ARHGAP26	7	54231683	0.833	A	G	9	0.222	9	intron_variant	MODIFIER	ENSBTAG00000027151	rs42350004
*EBF1**	7	70585950	0.063	G	T	8	0.833	9	intron_variant	MODIFIER	ENSBTAG00000037508	
*EBF1**	7	70585950	0.875	G	C	8	0.111	9	intron_variant	MODIFIER	ENSBTAG00000037508	rs41656037
FAM129A	16	66006047	0.25	A	G	8	0.889	9	intron_variant	MODIFIER	ENSBTAG00000014548	rs41817426
WIPI1	19	61782120	0.25	T	C	8	0.889	9	downstream gene variant	MODIFIER	ENSBTAG00000010637	rs41928661
*ARSG**	19	61782120	0.25	T	C	8	0.889	9	intron_variant	MODIFIER	ENSBTAG00000039556	rs41928661
*ARSG**	19	61874326	0.813	C	A	8	0.167	9	intron_variant	MODIFIER	ENSBTAG00000003074	rs450146987
SLC16A6	19	61874326	0.813	C	A	8	0.167	9	intron_variant	MODIFIER	ENSBTAG00000010637	rs450146987
PFKFB4	22	51327126	0.938	A	G	8	0.222	9	intron_variant	MODIFIER	ENSBTAG00000006752	rs42013635
BOLA-DOA	23	7314809	0.063	T	G	8	0.889	9	downstream gene variant	MODIFIER	ENSBTAG00000006490	Not reported
CCND3	23	15736092	1	T	C	8	0.056	9	intergenic_variant	MODIFIER	Not reported	rs110778610
